# Perioperative Outcomes Using Single-Fire Stapler

**DOI:** 10.1007/s11695-024-07357-4

**Published:** 2024-08-06

**Authors:** Gregory D. Fritz, Aryana Sharrak, Jason Aubrey, Xhesika Topalli, Antonia Vrana, Anne Opalikihn, Giuseppe M. Zambito, Thomas D. Martin, James A. Foote, Joshua R. Smith, Jon L. Schram

**Affiliations:** 1 Corewell Health – Grand Rapids/Michigan State University General Surgery Residency, Corewell Health, Grand Rapids, USA; 2 Michigan State University College of Human Medicine, Grand Rapids, USA; 3 Corewell Health Department of General Surgery, 1900 Wealthy St SE, Suite 250, Grand Rapids, MI 49506 USA; 4 Corewell Health Department of Bariatric Surgery, Grand Rapids, USA

**Keywords:** Sleeve gastrectomy, Stapling device, Bariatric surgery

## Abstract

**Background:**

Laparoscopic sleeve gastrectomy (LSG) is the most common bariatric surgery performed worldwide. The Titan stapler aims to standardize the sleeve gastrectomy by eliminating inconsistencies and simplifying the procedure.

**Methods:**

A retrospective chart review was performed on all patients > 18 years of age undergoing LSG using the Titan. Pre-operative demographics, perioperative findings, and post-operative complications were all abstracted from the MBSAQIP database.

**Results:**

A total of 807 LSG have been performed using the latest iteration of the Titan stapler since November 2022. Data from these patients was compared to 3829 patients who underwent LSG using a sequential staple firing technique from September 2016–September 2021. The median age of Titan patients was 42 years (IQR 33–52) compared to 44 years (IQR 35–54) for sequential firing. The median pre-operative BMI was 47.1 (IQR 43.5–52.1) for Titan versus 47.6 (IQR 43.1–53.3) for sequential staple firing. After propensity matching, operative duration was significantly less for the Titan. Titan patients had decreased hospital length of stay, experienced fewer 30-day readmissions, and had less post-operative nausea/vomiting. Post-op bleed rates were similar between the two cohorts. Weight loss at 6 months favored the sequential fire arm, but our preliminary data shows this difference diminishes at 1 year.

**Conclusions:**

Here we report our data on patients undergoing LSG using the latest Titan stapler. We show the device is safe, effective, and has resulted in an improvement in length of stay, readmissions, and post-operative nausea/vomiting. We also noted reduced operative time with this technique.

**Graphical Abstract:**

**Figure:**
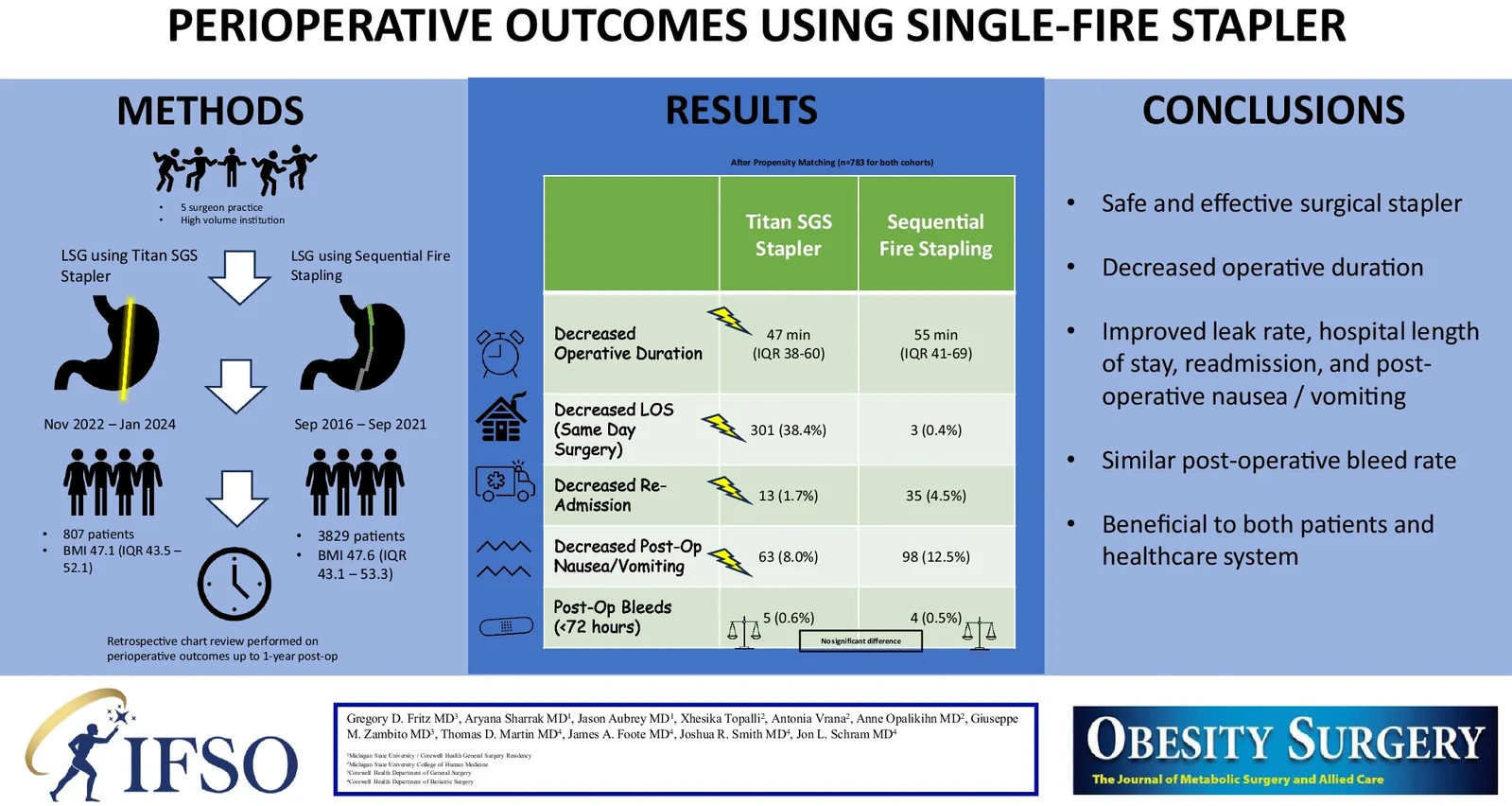

**Supplementary Information:**

The online version contains supplementary material available at https://doi.org/10.1007/s11695-024-07357-4.

## Introduction/Purpose

Obesity affects 42% of adults in the United States and has been shown to cost the healthcare system $173 billion annually [1–3]. Metabolic bariatric surgery is highly effective in the management of obesity and is indicated for patients with a BMI ≥ 35 or BMI 30 to 34.9 with type 2 diabetes [4]. The American Society for Metabolic and Bariatric Surgery (ASMBS) estimated that only 228,000 people undergo bariatric surgery annually, less than 1% of the potential surgical candidates [5]. Most surgeons suspect that fear of the operation is the rate-limiting step and that a very safe and effective surgery would increase the number of patients opting for surgical treatment. Common procedures in the United States include sleeve gastrectomy, Roux-en-Y gastric bypass (RYGB), and biliopancreatic diversion.

Laparoscopic sleeve gastrectomy (LSG) is a proven modality for effective weight loss and is the most commonly performed surgery for obesity in the United States and the world. LSG has many advantages in comparison with RYGB such as its technical simplicity, fewer anastomoses or foreign bodies, lower 30-day major morbidity (2.3% vs 4.4%, adjusted odds ratio (AOR) 0.53; *p* < 0.01), and lower 30-day mortality (0.2% vs 0.1%, AOR 0.58; *p* = 0.07) [6–9]. Complications associated with LSG include hemorrhage, staple-line leaks, post-operative gastroesophageal reflux disease, and stenosis [10]. In our experience, the success of LSG and the frequency of complications reflects the accuracy and efficiency of the surgical stapling process. The creation of a tubular-shaped sleeve without a twist or narrowing at the incisura has been shown to produce more favorable outcomes [11]. Much of the variability and decision-making intraoperatively involves the staple line. This variability stems from the thickness of the stomach in separate regions and the staple height utilized. As a result, different staple loads are used, and each sequential firing must be appropriately aligned with those prior in order to prevent twisting of the sleeve. Staple line leaks and bleeds are two of the feared immediate complications from the procedure leading many surgeons to utilize adjuvant modalities such as applying topical hemostatic agents or buttressing the staple line. An instrument that creates a sleeve without twist and is safe and effective in creating a reproducible gastric sleeve therefore seems prudent.

The Titan SGS stapler was developed by Standard Bariatrics (Cincinnati, Ohio, USA) and gained FDA approval in 2021. It is a powered, 23-cm single-fire stapler that employs staples of varying height (1.2 to 2.2 mm) to produce a straight and reproducible gastric sleeve in one fire, as seen in Animation 1. The Titan provides equal, uninterrupted compression along the entirety of the staple line, and fires staples from proximal to distal. As a result, the alignment of the stapling is ensured at the most critical portion of the staple line where leaks typically occur [12]. The Titan stapler’s proposed benefits include reduced operative time, reduced staple loads, removal of staple load junction sites, and eliminating potential angulation between staple loads. It is also proposed that a single-fire staple line will reduce unwanted twisting and narrowing at the incisura. Salyer et al. completed a multi-center pilot study using the Titan stapler on 61 patients to assess surgeon feedback and perioperative outcomes at 6 weeks. All surgeons were able to complete the sleeve gastrectomy with the Titan stapler and only one patient (1.6%) had a post-operative complication of hemorrhage requiring reoperation [11].

This retrospective study aims to elucidate the perioperative outcomes of LSG cases performed using the Titan stapler in direct comparison with sequential staple firing in one of the largest case series known to date regarding the Titan. We aim to assess patient outcomes with the stapler, and further demonstrate that it is a safe, effective tool, and improves efficiency in performing laparoscopic sleeve gastrectomy.

## Methods

We undertook an observational study on patients undergoing LSG using the latest iteration of the Titan stapler from November 2022 to January 2024 within a single high-volume institution with five experienced surgeons compared directly to a cohort of patients undergoing LSG performed by the same surgeons from September 2016 to September 2021 in which sequential staple firing was utilized. We used verified Metabolic and Bariatric Surgery Accreditation and Quality Improvement Program (MBSAQIP) data uploaded to the SET Collaborative database.

The study was determined to be exempt after review by the Institutional Review Board (#2022–128). Inclusion criteria were ≥ 18 years old and the patient underwent LSG. Exclusion criteria were procedure was converted to open, patient was lost to follow-up, or the patient had undergone previous bariatric surgery. Pre-operative demographics, and relevant perioperative data including adjuvant therapies used for the staple line, post-operative course, and complications were also obtained from the SET collaborative data. The analysis data consisted of 807 patients whose LSGs were done using the updated Titan stapler from November 2022 until January 2024. Cases done using the first-generation stapler, which is no longer on the market, were excluded. It also included 3829 patients who underwent LSG with the sequential firing method at the same institution during the 5 years prior to November 2022 (Table 1).

**Table 1:** Comparison of pre-operative patient characteristics (before and after propensity matching)

**Categorical variable**	Unmatched				Matched 1:1			
	Sequential firing (*n* = 3829)	Titan (n = 807)	*p*-value	95% CI for difference	Sequential firing (*n* = 783)	Titan (*n* = 783)	*p*-value	95% CI for difference
Female	2944 (76.8%)	629 (77.9%)	0.55	(− 0.04, 0.02)	610 (77.9%)	610 (77.9%)	Exact match forced	
Race			< 0.01	N/A			Exact match forced	
Black	358 (9.4%)	61 (7.65%)			61 (7.8%)	61 (7.8%)		
Other	97 (2.5%)	93 (11.5%)			72 (9.2%)	72 (9.2%)		
White	3374 (88.1%)	653 (80.9%)			650 (83.0%)	650 (83.0%)		
ASA class			< 0.01	N/A			Exact match forced	
1–2	316 (8.3%)	37 (4.6%)			37 (4.7%)	37 (4.7%)		
3	3372 (88.1%)	736 (91.2%)			718 (91.7%)	718 (91.7%)		
4	141 (3.7%)	34 (4.2%)			28 (3.6%)	28 (3.6%)		
Smoker	414 (10.8%)	54 (6.7%)	< 0.01	(0.02, 0.06)	62 (7.9%)	54 (6.9%)	0.32	(− 0.02, 0.04)
Diabetes at baseline	1084 (28.3%)	175 (21.7%)	< 0.01	(0.03, 0.10)	180 (23.0%)	168 (21.5%)	0.39	(− 0.03, 0.06)
GERD at baseline	1651 (43.1%)	293 (36.3%)	< 0.01	(0.03, 0.10)	302 (38.6%)	286 (36.5%)	0.35	(− 0.03, 0.07)
Hyperlipidemia at baseline	1120 (29.3%)	182 (22.6%)	< 0.01	(0.03, 0.10)	183 (23.4%)	178 (22.7%)	0.73	(− 0.04, 0.05)
Hypertension at baseline	1943 (50.7%)	340 (42.1%)	< 0.01	(0.05, 0.12)	325 (41.5%)	335 (42.8%)	0.37	(− 0.06, 0.04)
Sleep apnea at baseline	1500 (39.2%)	309 (38.3%)	0.66	(− 0.03, 0.05)	311 (39.7%)	299 (38.2%)	0.52	(− 0.03, 0.06)
History of DVT	195 (5.1%)	36 (4.5%)	0.53	(− 0.01, 0.02)	37 (4.7%)	35 (4.5%)	0.80	(− 0.02, 0.02)
History of PE	94 (2.5%)	23 (2.9%)	0.54	(− 0.02, 0.01)	25 (3.2%)	22 (2.8%)	0.87	(− 0.01, 0.02)
Therapeutic anticoagulation	162 (4.2%)	38 (4.7%)	0.57	(− 0.02, 0.01)	37 (4.7%)	38 (4.9%)	0.90	(− 0.02, 0.02)
**Continuous variable**	Unmatched				Matched			
	Sequential firing (*n* = 3829)	Titan (*n* = 806)	*p*-value	95% CI for shift	Sequential firing (*n* = 783)	Titan (*n* = 783)	*p*-value	95% CI for shift
	Median (IQR)				Median (IQR)			
Age at baseline	44 (35–54)	42 (33–52)	< 0.01	(− 3, − 1)	42 (33–52)	43 (33–52)	0.57	(− 2, 1)
BMI at baseline	47.6 (43.1–53.3%)	47.1 (43.5–52.1)	0.44	(− 0.7, 0.3)	47.1 (43.0–52.8)	47.1 (43.4–52.1)	0.70	(− 0.5, 0.8)

Complications were extracted from chart review extending up to 1 year post-operatively and followed previously defined MBSAQIP definitions. Complications included post-operative bleed, confirmed either via abdominal re-exploration or suspected with significant drop in hemoglobin requiring transfusion. Post-operative leak was included if evident on post-operative imaging study or during re-exploration. Post-operative stricture was identified on post-operative imaging or endoscopic evaluation.

Categorical patient demographic variables and comorbidities were reported as counts and percentages. They were compared between groups using exact Fisher tests, risk differences, and associated 95% confidence intervals were also calculated. Because of skewed distributions, continuous pre-operative variables were reported as medians and interquartile ranges. They were compared using Wilcoxon rank sum tests; Hodges-Lehmann estimates of distributional shifts were calculated along with their 95% confidence intervals.

Because statistically significant differences were detected for some predictors, we undertook propensity score matching. SAS PROC PSMATCH was used for greedy matching in the region of common support. Exact matches were requested for patient sex, race, and ASA scores. We then used McNemar, Bhapkar, and signed rank tests to check that all pre-operative differences had been adjusted away. We then compared outcomes between groups, again using McNemar and signed rank tests for the matched pairs. Analysis was carried out using SAS 9.4. The statistical significance value was set at *p* = 0.05.

## Operative Technique

A similar operative technique and instruments were utilized for all patients. All procedures were performed either laparoscopic or robotic assisted.

All sequential firing LSGs were performed using the Echelon 60-mm powered stapler (Ethicon™ Biosurgery, Inc., Somerville, NJ, USA) over a 38–40 French bougie (ViSiGi 3D™, Boehringer Labs, LLC, Phoenixville, PA, USA). Once the greater curve dissection was completed, the first two staple firings were typically performed using black staple loads, followed by an additional 3–4 green staple loads until reaching the Angle of His, and the entire specimen was resected. All staple loads were fired with buttressing material in place (SEAMGUARD® (GORE®; W.L. Gore & Associates, Elkton, MD, USA) or Peri-Strips® (Baxter Healthcare Corp., Deerfield, IL, USA)). In all cases, the proximal 5–10 cm of the staple line was oversewn with 2–0 Vicryl (Ethicon™ Biosurgery, Inc., Somerville, NJ, USA) or Stratafix (Ethicon™ Biosurgery, Inc., Somerville, NJ, USA) securing omentum to the staple line. Topical hemostatic agents were utilized on a case-by-case basis at the discretion of the surgeon.

For LSG using the Titan stapler, once the greater curve dissection was completed, the stapler was introduced via a 19-mm trocar placed to the right of the umbilicus approximately 27 cm inferior to the xiphoid process. The stapler was fired over a 38 French ballooned bougie (Teleflex Inc., Wayne, PA, USA) filled with 14 cc of saline, from 5 to 6 cm proximal to the pylorus along the greater curve ending at the Angle of His. The staple line is then similarly managed with over-sewing and topical hemostatic agent application. The 19-mm port site was closed in all cases to prevent an incisional hernia.

## Results

For the Titan stapler arm, the median patient age was 42 years (IQR 33–52) as compared with 44 years (IQR 35–54) for the sequential staple fire arm. The difference was statistically significant (*p* < 0.01). A total of 629 patients were female (77.9%) and 178 were male. The median initial BMI was 47.1 (IQR 43.5–52.1) in the Titan arm compared to 47.6 (IQR 43.1–53.3) in the sequential staple fire arm, thus demonstrating BMI equivalence between the two groups.

To summarize, there were detectable differences between treatment groups for patient age at surgery as well as for race, ASA score, and smoking history. To adjust for these differences, we calculated propensity scores and used them to create matched cohorts. Using the matching procedure in SAS, we were able to match each of the 783 (97%) Titan patients to a unique sequential staple firing patient. We required exact matching on sex, race, and ASA score. Table 1 demonstrates that propensity matching eliminated all statistically significant differences between groups.

The matched cohorts were then used to assess operative outcomes. As indicated in Table 2, we found that the median operative duration for sequential staple firing LSG was 55 min (IQR 41–69), and for the Titan, it was 47 min (IQR 38–60, *p*-value < 0.01). The median length of stay was 1 day for sequential staple firing and 1 day for Titan, although the narrower IQR for the Titan arm implied a significant difference (*p* < 0.01) between patient groups along with increased consistency. Perioperative complications, readmissions, and reoperation data are also reported in Table 2. Titan patients experienced fewer 30-day readmissions, especially those related to nausea/vomiting. The proportions of reoperations and leaks by 30-days were favorable to the Titan arm but did not reach statistical significance. Post-operative bleeds within 72 h were similar between the two cohorts. Sequential staple fire patients had 1–2% improved weight loss at 6 months following surgery. No post-operative port site hernias have been encountered to date. No instrument misfires or malfunctions have been encountered to date.

**Table 2:** Perioperative outcomes comparison using propensity-matched data (783 matched pairs)

**Categorical variable**	Sequential firing		Titan			*p*-value	95% CI for difference
Bleed by 72 h	4 (0.5%)		5 (0.6%)			0.74	(− 0.01, − 0.00)
Leak by 30 d	4 (0.5%		0 (0.0%)			< 0.02	(0.00, 0.01)
Readmission by 30 d	35 (4.5%)		13 (1.7%)			< 0.01	(0.01, 0.05)
Reoperation by 30 d	8 (1.0%)		3 (0.4%)			0.13	(− 0.00, 0.01)
Stricture by 30 d	2 (0.3%)		0 (0.0%)			–-	(− 0.0, 0.01)
Intervention by 30 d	13 (1.7%)		0 (0.0%)			–-	(− 0.03, − 0.01)
N/V and related by 30 d	83 (10.6%)		72 (9.2%)			0.36	(− 0.04, 0.02)
Organ space SSI	6 (0.8%)		1 (0.1%)			0.06	(− 0.01, 0.00)
Deep incisional SSI	0 (0.0%)		0 (0.0%)			–-	–-
Superficial incisional SSI	12 (1.5%)		5 (0.6%)			0.09	(− 0.01, − 0.02)
LOS						< 0.01	N/A
Same day	3 (0.4%)		301 (38.4%)				
1 day	506 (64.6%)		401 (51.2%)				
2–7 days	269 (34.4%)		81 (10.3%)				
> 7 days	5 (0.6%)		0 (0.0%)				
**Continuous variable**	Sequential Firing			Titan			
	Median (IQR)	Mean (S.D.)		Median (IQR)	Mean (S.D.)	*p*-value	95% CI for shift
OR time (min.)	55 (41–69)	59 (24)		47 (38–60)	51 (19)	< 0.01	(4.0, 7.0)
% weight loss to 6 m	22 (19–27)	23 (7)		21 (17–25)	21 (6)	< 0.01	(− 2.7, − 0.7)

## Discussion

LSG has become the most commonly performed surgical procedure for the treatment of obesity and related metabolic disorders worldwide. LSG is a restrictive and metabolic operation in which approximately 80% of the stomach is removed leaving behind a tubular gastric sleeve. Short-term follow-up after LSG has demonstrated good weight loss, remission of associated medical problems, and a minimal number of patients with weight regain and gastroesophageal reflux disease [13]. Variability in how the procedure has traditionally been performed revolves around the size and number of staple loads utilized, and techniques implemented to reinforce the staple line.

Multiple staple firings and reloads can be time-consuming, increase the risk of complications, and may lead to suboptimal surgical outcomes as noted by the FDA [14]. The FDA has reported an increased leak rate with stapling tissues outside the maximum and minimum tissue thickness limits for the stapler, as well as with overlapping staple lines. Multiple staple firings can also lead to twisting of the sleeve and unwanted narrowing at the incisura [11]. To address these challenges, the Titan single-fire stapler was designed specifically for LSG. Previous studies have demonstrated the stapler to be both safe and effective [12].

Staple line leaks are a feared complication of LSG and typically occur just below the level of the gastroesophageal junction. These can be difficult to resolve given the higher intragastric pressure and contents involved [7]. Leak rates reported in the literature occur between 1.7 and 4.9% of cases [6]. Sleeve leaks can result in a significant burden to both the patient and hospital system. Older age and higher BMI have both been linked with higher leak rates [15–17]. Other risk factors including surgeon experience, size of bougie, distance from the pylorus, the presence of narrowing at the incisura, and staple line reinforcements have all been previously discussed in the literature [6, 15–19]. A systematic review from 2018 by Gagner et al. analyzed the use of five separate staple line reinforcement methods including no reinforcement, over-sewing, bovine pericardial membrane, tissue sealant, and absorbable polymer membrane among 40,653 patients. The use of an absorbable polymer membrane was shown to have the lowest leak rate at 0.39% (9/2302) and leak rates were comparable between the use of Tisseel® and performing no reinforcement at all [6]. Omentopexy is an additional method for reinforcement of the staple line, wherein the greater omentum is re-secured to the gastric staple line using an absorbable suture. There is ongoing debate in the literature that the technique reduces rates of volvulus and obstruction whilst improving post-operative nausea and vomiting. The method however is safe, requires minimal additional operative time, and has been shown to decrease gastric sleeve leak rates [20].

Gastric sleeve shape using a sequential stapling technique can be quite variable, which has post-operative consequences. Producing a straight, tubular sleeve is the end goal. Previous studies by Toro et al. evaluated outcomes associated with variations in gastric sleeve morphology. Although no difference in weight loss was identified among the four separate sleeve sub-types (tubular, upper pouch, lower pouch, dumbbell), reflux and hunger symptoms varied significantly in those with non-tubular gastric sleeves [11]. Additionally, twisting of the sleeve, primarily the result of staple loads fired in varying anterior/posterior planes, exposes patients to reflux, dysphagia, and at extremes can result in obstruction [21]. We believe creating a straight tubular sleeve plays a large role in post-operative recovery, as demonstrated by the statistically significant differences in both 30-day readmissions and reported post-operative nausea and vomiting.

In our study, 807 LSG procedures using the latest iteration of the Titan stapler were performed from November 2022 to January 2024. The median patient age was 42 years (IQR 33–52), and median initial BMI was 47.1 (IQR 43.5–52.1) for the Titan arm. 77.9% of Titan sleeve patients were female. Using the Titan, 0 staple line leaks were encountered out of 807 cases performed. Our reported leak rate is less than the rate we observed when using a sequential fire method.

Post-operative bleeds following LSG are reported to occur in 1.1–8.7% of cases [6]. It is often difficult to determine the exact site of the bleeding, but the gastric staple line and the gastroepiploic or short gastric vessels are often suspected. Similar to gastric staple line leaks, bleeds can relate to surgeon experience in addition to the staple load utilized. Incorrect staple height results in inappropriate compression/apposition during the stapling process leaving exposed submucosa that can bleed. Staple line reinforcement techniques result in decreased post-operative bleeding complications, as shown by numerous studies [19]. Our study encountered 5 post-operative bleeds out of the 807 cases performed. Four patients were treated conservatively with no additional interventions required. One patient required re-operation for washout. The location of the bleeds in all five cases was not identified. Our reported post-operative bleed rate of 0.6% is similar to rates published in the literature.

Operative time was significantly less when the Titan stapler was used compared to the sequential fire technique. Surgeon experience could influence the performance of LSG. However, the same experienced surgeons performed the procedures for both cohorts of patients, thereby reducing the operative experience bias. The Titan stapler takes approximately 1 to 2 min for firing to complete, varying based on tissue thickness and compression time. Although average operative time differed by 8 min between the two groups, additively this results in more cases that can be performed daily and further displays the utility of the instrument. Weight loss at 6 months was slightly yet significantly greater in the sequential fire group. Our preliminary review of the 1-year data shows that this difference diminishes. We postulate that a straighter staple line without twists may reduce the number of patients who have very rapid weight loss associated with nausea, vomiting, and dysphagia related to malformed sleeves. This observation is bolstered by a lower readmission rate attributed to nausea/vomiting for Titan versus sequential firing.

There are both advantages and disadvantages with each approach for LSG. The Titan stapler requires 19-mm port site which must be closed to prevent a post-operative hernia. However, the larger port site allows for easy removal of the specimen without additional expansion. The Titan stapler is also not approved for LSG in cases for which the patient has undergone previous gastric procedural intervention. The utilization of buttressing and topical hemostatic agent application to the staple line may contribute minimally to the overall outcome. They are utilized in our practice as a replacement for SEAMGUARD®/Peri-Strips® which were not available on the Titan version used in this study. The use of the Titan stapler allows for a reproducible technique with no overlapping staple lines, and based on our observations, produces a higher rate of tubular gastric sleeve without twist compared with our sequential fire technique. Lastly, the use of the Titan stapler is quicker in comparison to sequential staple firing.

## Conclusions

Here we report our data on patients undergoing LSG using the latest iteration of the Titan single-fire stapler since its implementation in our practice. We again demonstrate similar post-operative bleed rates to the literature but show improvement in post-operative leaks. A total of 807 patients have since undergone LSG using the Titan stapler, making this the largest cohort of patients published to date. The simplified and efficient stapling process offered by this instrument has the potential to enhance the safety and effectiveness of LSG, ultimately benefiting patients and healthcare providers. We have provided evidence that the device is safe and effective and may result in improved outcomes regarding length of stay, readmission, reoperation, and stricture formation. In addition, we have documented considerable time savings with this technique. Further studies will be done to determine its ability to reduce post-operative reflux which we have noticed anecdotally but have not investigated further.

## Supplementary Information

Below is the link to the electronic supplementary material.

Supplementary file1 (MP4 405 MB)

## References

[1] Ward ZJ, Bleich SN, Long MW, et al. Association of body mass index with health care expenditures in the United States by age and sex. PLoS ONE. 2021;16(3):e0247307. 10.1371/journal.pone.0247307. PMC7990296 33760880

[2] Barski K, Binda A, Kudlicka E, et al. Gastric wall thickness and stapling in laparoscopic sleeve gastrectomy - a literature review. Wideochir Inne Tech Maloinwazyjne. 2018;13(1):122–7. 10.5114/wiitm.2018.73362. PMC5890851 29643968

[3] Jensen MD, Ryan DH, Apovian CM, et al. 2013 AHA/ACC/TOS guideline for the management of overweight and obesity in adults: a report of the American College of Cardiology/American Heart Association Task Force on Practice Guidelines and The Obesity Society. Circulation. 2014;129(25 Suppl 2):S102-138. 10.1161/01.cir.0000437739.71477.ee. PMC5819889 24222017

[4] Who is a Candidate for Bariatric Surgery? | Patients | ASMBS. American Society for Metabolic and Bariatric Surgery. Accessed September 27, 2023. https://asmbs.org/patients/who-is-a-candidate-for-bariatric-surgery.

[5] Estimate of Bariatric Surgery Numbers, 2011–2021. American Society for Metabolic and Bariatric Surgery. Published June 27, 2022. Accessed September 27, 2023. https://asmbs.org/resources/estimate-of-bariatric-surgery-numbers.

[6] Gagner M, Kemmeter P. Comparison of laparoscopic sleeve gastrectomy leak rates in five staple-line reinforcement options: a systematic review. Surg Endosc. 2020;34(1):396–407. 10.1007/s00464-019-06782-2. PMC6946737 30993513

[7] Gagner M, Cardoso AR, Palermo M, et al. (editors). The perfect sleeve gastrectomy: a clinical guide to evaluation, treatment, and techniques. Springer International Publishing. 2020. 10.1007/978-3-030-28936-2.

[8] Palermo M, Gagner M. Why we think laparoscopic sleeve gastrectomy is a good operation: step-by-step technique. J Laparoendosc Adv Surg Tech A. 2020;30(6):615–8. 10.1089/lap.2020.0154. 32319850

[9] Singhal R, Cardoso VR, Wiggins T, et al. 30-day morbidity and mortality of sleeve gastrectomy, Roux-en-Y gastric bypass and one anastomosis gastric bypass: a propensity score-matched analysis of the GENEVA data. Int J Obes. 2022;46:750–7. 10.1038/s41366-021-01048-1. PMC8671878 34912046

[10] Trastulli S, Desiderio J, Guarino S, et al. Laparoscopic sleeve gastrectomy compared with other bariatric surgical procedures: a systematic review of randomized trials. Surg Obes Relat Dis. 2013;9(5):816–29. 10.1016/j.soard.2013.05.007. 23993246

[11] Toro JP, Lin E, Patel AD, et al. Association of radiographic morphology with early gastroesophageal reflux disease and satiety control after sleeve gastrectomy. J Am Coll Surg. 2014;219(3):430–8. 10.1016/j.jamcollsurg.2014.02.036. 25026879

[12] Salyer C, Spuzzillo A, Wakefield D, et al. Assessment of a novel stapler performance for laparoscopic sleeve gastrectomy. Surg Endosc. 2021;35(7):4016–21. 10.1007/s00464-020-07858-0. 32749610

[13] Deitel M, Crosby RD, Gagner M. The first international consensus summit for sleeve gastrectomy (SG), New York City, October 25–27, 2007. Obes Surg. 2008;18(5):487–96. 10.1007/s11695-008-9471-5. 18357494

[14] General and Plastic Surgery Devices; Reclassification of Certain Surgical Staplers. Federal Register. Published October 8, 2021. Accessed March 24, 2024. https://www.federalregister.gov/documents/2021/10/08/2021-22041/general-and-plastic-surgery-devices-reclassification-of-certain-surgical-staplers.

[15] Aurora AR, Khaitan L, Saber AA. Sleeve gastrectomy and the risk of leak: a systematic analysis of 4,888 patients. Surg Endosc. 2012;26(6):1509–15. 10.1007/s00464-011-2085-3. 22179470

[16] Comparative use of different techniques for leak and bleeding prevention during laparoscopic sleeve gastrectomy: a multicenter study - PubMed. Accessed September 27, 2023. https://pubmed.ncbi.nlm.nih.gov/24448100/. 10.1016/j.soard.2013.10.018 24448100

[17] Berger ER, Clements RH, Morton JM, et al. The impact of different surgical techniques on outcomes in laparoscopic sleeve gastrectomies: the First Report from the Metabolic and Bariatric Surgery Accreditation and Quality Improvement Program (MBSAQIP). Ann Surg. 2016;264(3):464–73. 10.1097/SLA.0000000000001851. 27433904

[18] Varban OA, Sheetz KH, Cassidy RB, et al. Evaluating the effect of operative technique on leaks after laparoscopic sleeve gastrectomy: a case-control study. Surg Obes Relat Dis. 2017;13(4):560–7. 10.1016/j.soard.2016.11.027. 28089439

[19] Cesana G, Cioffi S, Giorgi R, et al. Proximal leakage after laparoscopic sleeve gastrectomy: an analysis of preoperative and operative predictors on 1738 consecutive procedures. Obes Surg. 2018;28(3):627–35. 10.1007/s11695-017-2907-z. 28840492

[20] Zarzycki P, Kulawik J, Małczak P, et al. Laparoscopic sleeve gastrectomy with omentopexy: is it really a promising method? A systematic review with meta-analysis. Obes Surg. 2021;31(6):2709–16. 10.1007/s11695-021-05327-8. PMC8113139 33677783

[21] Salyer CE, Thompson J, Hanseman D, et al. Surprising neutral effect of shorter staple cartridges in laparoscopic sleeve gastrectomy. Surg Endosc. 2022;36(7):5049–54. 10.1007/s00464-021-08865-5. 34767062

